# Supplementary material for: Genome-wide analysis of lncRNA stability in human
Source: PLoS Comput Biol. 2021 Apr 16;17(4):e1008918. doi: 10.1371/journal.pcbi.1008918 (PMC8081339; doi:10.1371/journal.pcbi.1008918)
Supplement: S1 Text — (PDF) [file pcbi.1008918.s061.pdf]

METHOD DETAILS

1.1 Cell culture

Human Lung Adenocarcinoma Cells (A549) were incubated at 37°C and 5% CO_2 in a humidified atmosphere with DMEM medium (Sigma, 8119235) containing 10% fetal bovine serum (PAN, ST30-3302) and antibiotics (100 U/ml of penicillin and 0.1 mg/ml of streptomycin). For subcultivation, cells were washed with PBS, then incubated in the presence of trypsin/EDTA solution at 37°C for 1min. Medium was used to stop digestion. Then cells were plated into diameter 10cm culture dishes.

1.2 Inhibiting transcription

We used actinomycin D（Sigma, A1410）to inhibit transcription. Actinomycin D was dissolved by DMSO and the concentration is 1mg/ml. Using DMEM medium to dilute actinomycin D and the working concentration was 30μg/ml. We replaced DMEM medium with actinomycin D to inhibit transcription when cells reached 60%-70% confluency.

1.3 RNA extraction

Cells were harvested after inhibiting transcription with actinomycin D for 0, 0.5, 1, 1.5, 2, 3, 4, 5, 6 and 8h. Cells were washed for three times with PBS and

digested with trypsin/EDTA at 37°C for 1 min. The total RNA were purified with RNeasy Plus Mini kit (QiAGEN, 74134) as per manufacturer's instructions.

1.4 Separation of cytoplasm and nucleus

Cells were harvested after inhibiting transcription with actinomycin D for 0, 6, 12 and 24 h. All operations were done on ice. Cell membrane was lysed by 500μl lysate buffer (10mM Hepes-NaOH (pH7.9) 1ml, 10mM KCl 1ml,1.5mM $MgCl_2$ 150μl, β-Mercaptoethanol 3.5ml, protease inhibitor(Thermo, A32963) 1ml, phosphatase inhibitor (Thermo, A32957) 10ml and add DEPC water calibrating to 100ml) and RNase Inhibitor (Takara, 2313A) in EP tube. The cells were lysed on a spinning wheel for 45min at 4°C. For getting the cytoplasm, the tubes were high-speed centrifugation(8200r/min) for 5min at 4 ° C. Then, aspirating the supernatant (400μl) which is the cytoplasm. We washed the pellet with nucleus cleaning agent (1ml PBS, 10μl NP-40 and 5μl RNase inhibitor) ,then rotating on the spinning wheel for 5min and high-speed centrifugation (8200r/min) for 5min at 4°C. We removed the supernatant and repeated operations for three times. The precipitation is the nucleus.

1.5 Cytoplasm and Nucleus RNA extraction

We used TRI Reagent (Sigma, T3934) to extract cytoplasm RNA and Trizol (Sigma, 93289) to extract nucleus RNA. Nucleus or cytoplasm were first homogenized in Trizol or TRI Reagent and stand still for 5 minutes at room

temperature. Then, we added 200 μl chloroform and mixed well for 5 min. Samples were centrifuged (12000r/min) for 15 min at 4°C. We extracted the supernatant and added 500 μl isopropanol, then mixed well for 5min.Through centrifuging (12000r/min) for 10min at 4°C , we got the precipitation. Finally, we added 75% ethanol and centrifuged (7500r/min) for 5min at 4°C. RNA was dissolved by DEPC water and concentration was assessed by spectrophotometer.

1.6 qRT-PCR

We used qRT-PCR to assess the separation effect. We analyzed the RNA from NC group where cells were inhibited transcription for 0h. For getting the cDNA, we used 1μg RNA template, 10nM oligo-dT, and DEPC water, the total capacity is 5μl. Then proceed to 70°C metal bath for 5min and put on ice for 5min immediately. Then, we used ImProm-II^{TM} Reverse transcriptase (Promega, A3803) as per manufacturer's instructions. The sign primer of cytoplasm is GAPDH (F-TCAGTGGTGGACCTGACCTG, R-TGCTGTAGCCAAATTCGTTG) and the nucleus is Malat 1 (F-ATTCCGGTGATGCGAGTTGT, R-TCTGCGGTTTCCTCAAGCTC). We mixed 10μl SYBR™ Green PCR Master Mix(Thermo, 4309155)，8μl DEPC water,1μl cDNA and 1μl primer. Reaction conditions: Pre-denaturation 95°C for 3min, denaturation 95°C for 10 sec, annealing 60°C for 20 sec, 45 cycles. The result is shown in Fig S1, indicating that the separation method work well.

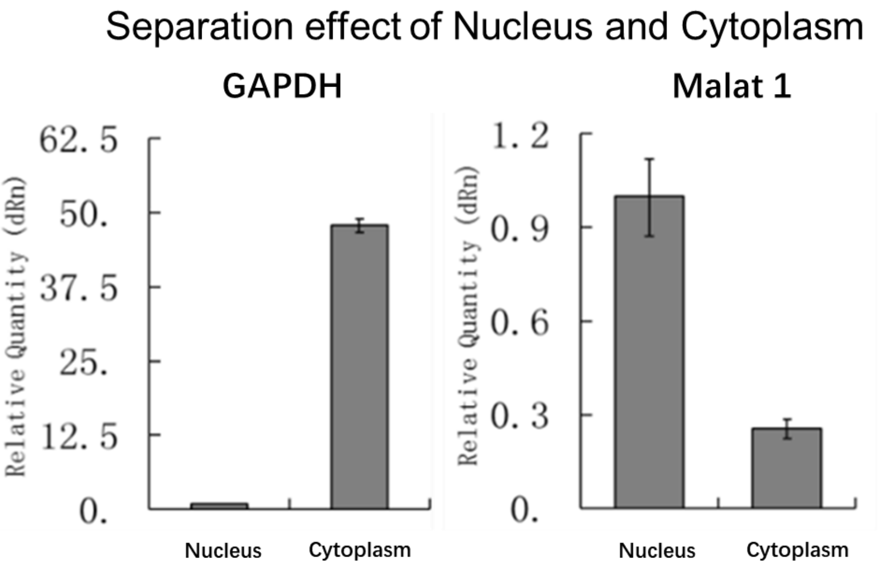

Fig S1. qRT-PCR showing GAPDH enriched in cytoplasm and Malat1 enriched in nucleus, indicating that nucleus and cytoplasm were separated well.

SUPPLEMENTAL REFERENCE

1. Shen Y, Liu S, Fan J, Jin Y, Tian B, Zheng X, Fu H: **Nuclear retention of the lncRNA SNHG1 by doxorubicin attenuates hnRNPC-p53 protein interactions**. *EMBO Rep* 2017, **18**(4):536-548.
