# Supplementary figures and images for: Genome-wide analysis of lncRNA stability in human
Source: PLoS Comput Biol. 2021 Apr 16;17(4):e1008918. doi: 10.1371/journal.pcbi.1008918 (PMC8081339; doi:10.1371/journal.pcbi.1008918)

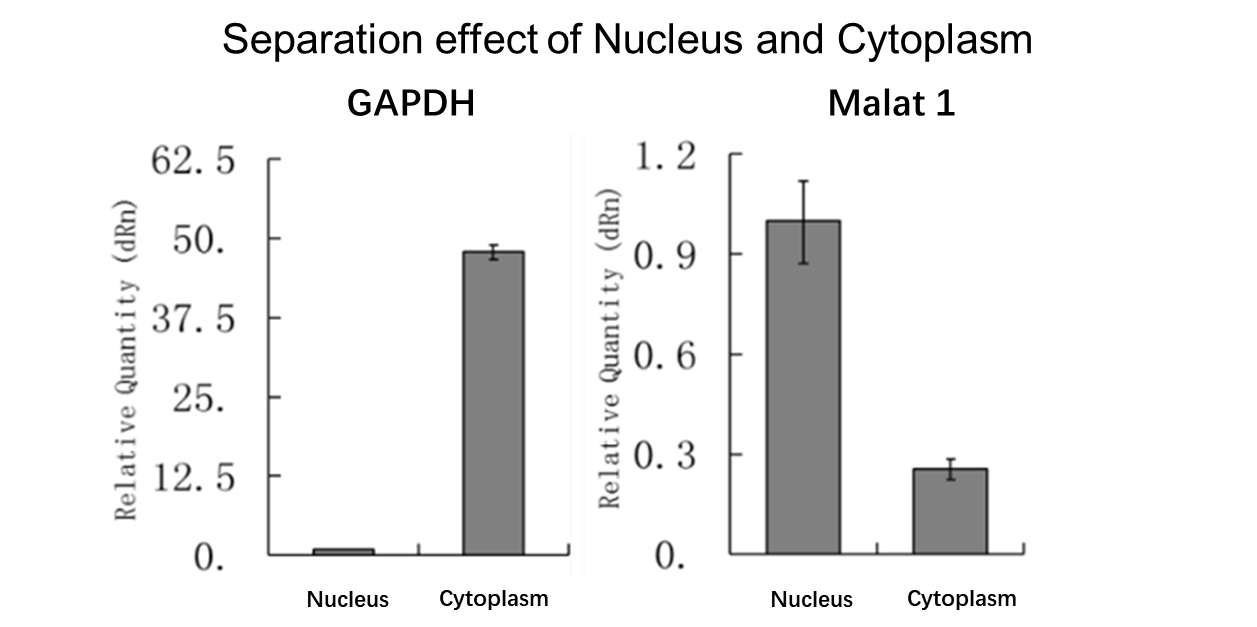

Supplement: S1 Fig — (TIF) [file pcbi.1008918.s062.tif]
