# Supplementary material for: Genome-wide analysis of lncRNA stability in human
Source: PLoS Comput Biol. 2021 Apr 16;17(4):e1008918. doi: 10.1371/journal.pcbi.1008918 (PMC8081339; doi:10.1371/journal.pcbi.1008918)
Supplement: Attachment — Submitted filename: Response 2.docx [file pcbi.1008918.s063.docx]

Dear Editor

Thank you very much for your valuable information to improve our manuscript. According to your suggestions, we have modified the related parts. The detailed modification information is as follows.

Best regards.

Yours sincerely

Wuju & Xiaofei

1. We note that the following authors have not yet confirmed their authorship on this manuscript: Tao Liu, Hanjiang Fu, xiaofei Zheng

Reply: We have asked them to confirm their authorship.

2. Please provide  separate figure files in .tif or .eps format only and remove any figures embedded in your manuscript file.  Please ensure that all files are under our size limit of 20MB.

Reply: First, we have removed all figures embedded in our revised manuscript. Second, each figure was separately provided in .tif format.

3. We have noticed that you have a list of supporting information legends in your manuscript. However, there are no corresponding files uploaded to the submission. Please upload them as separate files with the file type 'Supporting Information'. Please ensure that all Supporting Information files are included correctly and that each one has a legend listed in the manuscript

Reply: Sorry for this. Because of the large size of the supporting information including 60 files, we originally intended to provide a web page for users to download the data sets. However, after careful checking the data sets, we found that there are only two large data sets: Prediction-based miRNA-lncRNA interaction list and Prediction-based miRNA-mRNA interaction list. Therefore, in the revised version, we will upload the full information on the remaining 58 files. For the two large data sets, we will upload part information in the related files, in which we will provide a link to download the full information from our web page <http://ccb1.bmi.ac.cn:81/lncrnastability/.> The form is as following figure.

4. When completing the data availability statement of the submission form, you indicated that  "All supplementary datasets can be downloaded from our webpage http://ccb1.bmi.ac.cn:081/lncrnastability/.". However, you also checked the following box: “Tick here if the URLs/accession numbers/DOIs will be available only after acceptance of the manuscript for publication so that we can ensure their inclusion before publication.” If this box was checked in error, and there is no additional data to deposit, please ensure this box is unticked when you resubmit.

Reply: Thank you for your information. In the revised version, we have unticked the related option.

5. Please amend your Financial Disclosure statement to include any role the funders took in the study. If the funders had no role in your study, please state: “The funders had no role in study design, data collection and analysis, decision to publish, or preparation of the manuscript.

Reply: In the revised version, we have added the sentence “The funders had no role in study design, data collection and analysis, decision to publish, or preparation of the manuscript.” in the Financial Disclosure statement. Thanks for your information.
